# A Novel, Low-cost, Low-fidelity Pericardiocentesis Teaching Model

**DOI:** 10.5811/westjem.2021.3.49876

**Published:** 2021-07-19

**Authors:** Spencer Lord, Garrett Lord, Sean P. Geary

**Affiliations:** * Albany Medical Center, Department of Emergency Medicine and Department of Surgery, Albany, New York; † Dattner Architects, New York, New York

## Abstract

**Introduction:**

Pericardiocentesis is a high-risk/low-frequency procedure important to emergency medicine (EM). However, due to case rarity it is not often performed on a patient during residency training. Because the coronavirus disease 2019 pandemic limited cadaver-based practice, we developed a novel, low-cost, low-fidelity pericardiocentesis model using three dimensional-printing technology to provide advances on prior home-made models.

**Methods:**

Residents watched a 20-minute video about performing a pericardiocentesis and practiced both a blind and ultrasound-guided technique. We assessed model fidelity, convenience, and perceived provider competence via post-workshop questionnaire.

**Results:**

A total of 24/26 (93%) individuals practicing on the ultrasound-guided model and 22/24 (92%) on the blind approach model agreed or strongly agreed that the models reasonably mimicked a pericardial effusion.

**Conclusion:**

Our low-cost, low-fidelity model is durable, mimics the clinical case, and is easy to use. It also addresses known limitations from prior low-fidelity models.

## INTRODUCTION

Pericardiocentesis is a rare but potentially life-saving procedural intervention for release of pericardial fluid in unstable patients with cardiac tamponade. Historically performed by a subxiphoid approach using anatomical guidance in emergent cases, the procedure has now developed into an often ultrasound-guided (USG) procedure with increased success rate and fewer complications.^1^–^3^ Despite this improvement in management, the high-risk, low-occurrence nature of the procedure means providers can go prolonged periods of their career with minimal or no exposure including during their residency training. Furthermore, with the COVID-19 pandemic, residency programs have needed to find innovative ways to continue providing necessary medical education, as access to large-group, in-person, teaching situations and resources such as cadaver labs have been curtailed.^4^

Commercially developed models for pericardiocentesis are available but are often expensive with prices ranging in the several thousands of dollars.^5^,^6^ Furthermore, the use of high-fidelity models has not been shown to improve competency compared to low-fidelity alternatives, and a lack of true anatomical fidelity in the setting of functional fidelity also does not inhibit competency.^7^,^8^ Due to the need for proper training and the expense of high fidelity, a plethora of low-fidelity and home-made models have been made available.^9^–^15^ These models have also addressed common practical limitations such as using non-resin medium for ultrasonography and replaceable components. However, to the best of our knowledge, there are no low-fidelity models employing non-animal rib models.

To address this, we created an affordable pericardiocentesis model employing a low-cost, three dimensional (3D)-printed anatomical rib model from polylactic acid filament (PLA) that provides tactile feedback and appropriate interference during ultrasonography that can be generated with a personal 3D printer and software. The purpose of this study was to assess the feasibility of this model for training providers in both a blind approach (BA) and USG technique. Assessment of feasibility focuses on evaluating model fidelity, participant convenience, and participant-perceived competency.

## METHODS

### Basic Study Design

This was a prospective observational study performed at a single, Level I trauma center emergency medicine (EM) residency program between April–June of 2020. All study participants were EM residents between their first and third years of training. Each resident underwent a 20-minute preparatory session that reviewed both the BA and USG pericardiocentesis approaches using the following two videos: [https://www.youtube.com/watch?v=wKYWhutqzyg and https://www.youtube.com/watch?v=M4vHEr25yFk. Participants also watched a one-minute video on how to perform the procedure on the low-fidelity models. After this review, the participants then performed a BA and USG pericardiocentesis approach on two separate pre-made models. The procedures were performed independently to ensure safe, social distancing techniques during the COVID-19 pandemic. Upon completion, a survey was provided on site for participants to evaluate fidelity, convenience, and perceived competency for both approaches (see Supplemental).^14^ This study was determined to be exempt by the institutional review board as an anonymous survey and educational training project.

### Model Design

**Figure 1:**
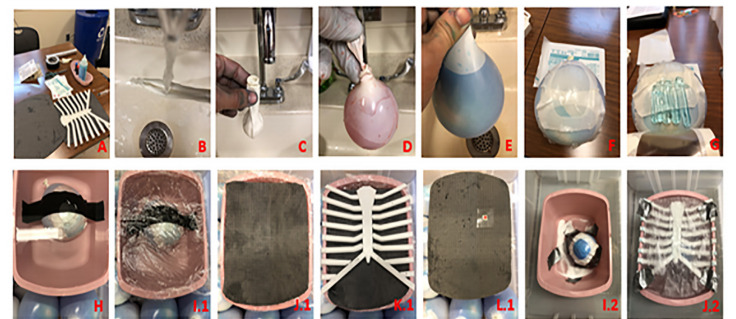
Design step-by-step progression: A. All supplies minus wash basin. B. Run warm water on the inner balloon over a blunt knife. C. Pass balloon inside outer balloon using knife. D. Place a drop of red food coloring and fill the inner balloon. E. Place a drop of blue food coloring and fill the outer balloon. F. Place a layer of Tegaderm on the outer balloon. G. Add a layer of ultrasound gel between layers of Tegaderm. H. Tape balloon to bottom of dry water basin. I.1. Place a layer of plastic wrap. J.1. Place the first layer of ¼″ yoga mat. K.1. Clip on anterior chest variant 1 to water basin. L.1. Place the second layer of ¼″ yoga mat and add the left shoulder indicator to the mat. I.2. Add polyethylene glycol (or equivalent). J.2. Tape a layer of parchment paper over the anterior chest variant 2 and fill the water top of basin.

**Figure 2:**
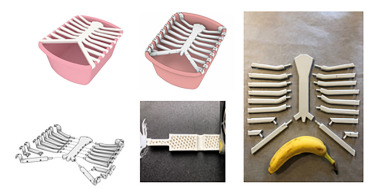
Clockwise: Blind approach pericardiocentesis anterior chest model; ultrasound-guided pericardiocentesis rendering; de-atomized model with scale; cross-section of printing lattice; and de-atomized rendering.

The pericardiocentesis model was constructed using materials found within an emergency department (ED) and personal home environment, along with a personal 3D printer and accompanying software. There are two model designs with interchangeable parts (Figure 1). Components include a seven-quart disposable plastic wash basin, red and blue food coloring, Tegaderm transparent film #1616 10 centimeters (cm) x 12 cm (3M, Minneapolis, MN,), 22.8 cm latex balloons, a Becton, Dickinson and Company spinal needle 18G x 3.5″ (BDC, Franklin Lakes, NJ), a Becton, Dickinson and Company 10-milliliter (mL) syringe Luer-Lok tip, duct tape, and tap water. For the BA pericardiocentesis model, additional materials were used including plastic wrap, parchment paper, and one yoga mat ¼″ extra thick deluxe. The USG model included Ultrasound Gel Aquasonic 100 transmission squeeze bottle (Parker Labs, Fairfield, NJ), archment paper roll, and Clearlax polyethylene glycol 3350 (Shopko Stores Operating Co., LLC, Greenbay, WI). We used a Creality Ender 3 3D printer (Creality Schenzhen, China) and 1.75 millimeter PLA filament (Hatchbox, Pomona, CA), and used Rhinoceros V6 software (McNeel and Associates, Seattle, WA, USA) for model construction and rendering (Figure 1). Project costs including modeling and rendering software are shown in Table 1. Digital development time for the two models took 10 hours. Prototyping, based on print, assembly, and revision, totaled 30 hours. A complete rendering of the finished anterior chest wall variants is shown in Figure 2. The GrabCAD link for printing details, BA model: https://grabcad.com/library/blind-pericardiocentesis-1; and USG Model: https://grabcad.com/library/ultrasound-guided-pericardiocentesis-1

### Workshop

Residents first independently reviewed a 20-minute video introducing the disease processes associated with pericardial effusions, as well as a video reviewing the two procedural approaches (landmark and ultrasound) with demonstrations on the current models. Participants then voluntarily signed up for rotating blocks of up to three people over rotating intervals to practice on the models. Finally, participants were asked to complete a short survey using a six-point Likert scale (5 strongly agree, 4 agree, 3 neutral, 2 disagree, 1 strongly disagree, n/a non-applicable) pertaining to questions regarding model fidelity, convenience, and competency. The workshop director was available for questions after independently attempting the model and questionnaire. The workshop director would also set up the model if the residents did not rebuild the model themselves.

### Blind Pericardiocentesis

**Figure 3:**
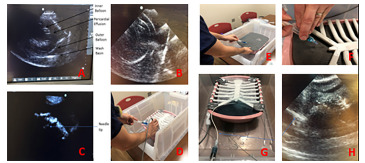
(A–D) Ultrasound-guided approach. (E–F) Blind approach. (G–H) Cannulation with pericardiocentesis kit.

This model was approached by identifying structurally equivalent anatomical landmarks of bony components of the anterior chest wall through physical exam. A small paper indicator was used to help participants orient caudad and cephalad. Aspiration was achieved using an 18-gauge lumbar needle attached to a 10 mL syringe (Figure 3).

### Ultrasound-guided Pericardiocentesis

This model was visualized using a polyethylene glycol solution as previously demonstrated by Sullivan et al (2018) using a Sonosite M-Turbo and a Sonosite MicroMaxx (FUJIFILM Sonosite, Inc., Bothell, WA) ultrasound machines.^14^ The goal was to identify an anechoic collection by either a subxiphoid or apical window approach between the two layers of latex balloons. This represented the pericardial effusion (Figure 3).

## RESULTS

During the study period 26/47 residents comprising 8/12 incoming interns during intern orientation week, 6/12 postgraduate year (PGY)-1s, 4/12 PGY-2s, and 8/11 PGY-3s completed the preparatory workshop, and used the task trainers; 26/27 consented to use their data for research purposes. Data analysis was performed with Excel 2006 (Microsoft Corp, Redmond, WA). For analyses, the incoming intern class and PGY-1 data were combined into the same PGY-1 category.

### Model Construction Practicality

Mean assembly time was 4.2 minutes for each model, based on the average production of six different models—3 BA and 3 USG approaches. Due to intentionally limiting the number of participants in the room, we did not calculate the average number of puncture attempts per model. The production time for the flat 3D printed chest model was 9 hours and 37 minutes. The production time for the 20 millimeter depressed model was 11 hours 3 minutes for the first print and 14 hours 24 minutes for the second print, totaling 25 hours 27 minutes.

### Model Feasibility

**Figure 4:**
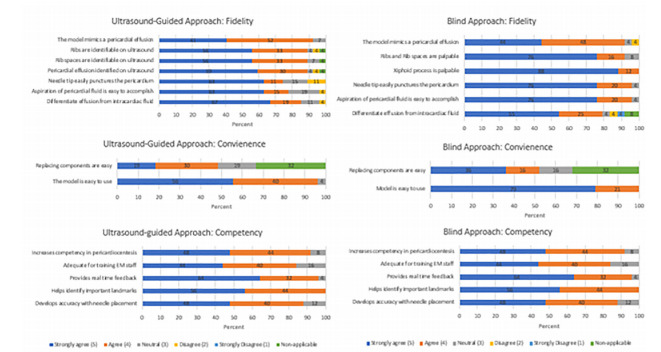
Results from the fidelity, convenience and competency questionnaire provided the participating residents. Values represent percentages. For the proportion of individuals that answered each subcategory refer to supplemental 2.

A total of 26/26 residents (14/14 PGY-1, 4/4 PGY-2, 8/8 PGY-3) completed the model for the USG pericardiocentesis model, and 24/26 (13/14 PGY-1, 4/4 PGY-2, 7/8 PGY-3) (92%) of the same residents completed the survey for the BA pericardiocentesis model. Regarding color of aspirate, 22/24 (13/13 PGY-1, 4/4 PGY-2, 5/7 PGY-3) (92%) commented on the first color aspirated during the BA with 18/22 aspirating blue (11/13 PGY-1, 4/4 PGY-1, 4/7 PGY-3) (82%), 3/22 (2 PGY-1, 1 PGY-2) (14%) aspirating red first, and one balloon rupture (PGY-1). For the USG model 23/26 (PGY-1 12/14, PGY-2 4/4, PGY-3 7/8) (88%) commented on the color aspirated with 19/26 aspirating blue (10/14 PGY-1, 4/4 PGY-2, 5/8 PGY-3), 3/26 aspirating a red color (one PGY-3, two PGY-1, and one balloon rupture (PGY-3). The frequency of distribution to responses are displayed in Figure 4.

### Fidelity

A total of 24/26 (92%) individuals practicing on the USG model and 22/24 (92%) from the BA model agreed or strongly agreed that the models mimicked a pericardial effusion. In the USG model, 23/26 (88%) agreed or strongly agreed that the ribs and ribs spaces were easily identifiable and in the BA model, 22/24 (92%) of participants agreed or strongly agreed that the ribs and rib spaces were palpable. Furthermore, 22/26 (85%) from the USG group and 23/24 (96%) from the BA strongly agreed or agreed that the aspiration of pericardial fluid was easily accomplished.

### Convenience

Regarding ease of use, 25/26 (96%) participants found the USG model easy to use, and 23/24 (96%) participants found the BA easy to use with one individual not answering.

### Competency

A total of 22/24 (92%) in the BA approach model and 21/25 (84%) in the USG group perceived that the training session increased their competency in pericardiocentesis.

## DISCUSSION

Here we describe a low-cost, low-fidelity model created with easy-to-purchase components. The rib and sternal mimics were easily constructed with an inexpensive 3D printer for performing a BA and USG pericardiocentesis (Table 1). In terms of cost the printer purchased for model production is one of the most affordable on the market compared to other available home models, and we included the acquisition cost in the overall cost of our model despite this being a one-time expense.^16^–^20^ Multiple high-quality, low-fidelity models have been published, and expensive high-fidelity US simulation trainers exist; however, none of the low-fidelity models provide practitioners with the associated physical exam and anatomical difficulties that are available in expensive, high-fidelity models without using animal products. A majority of the participants found the 3D-model rib structures and rib spaces easy to identify by physical palpation and by ultrasound. These findings are important because providers need to be comfortable with both the physical exam and ultrasound views, as well as being able to effectively aspirate an effusion. Thus, this model aptly prepares providers for navigating the whole procedure.

Our model was designed to make small advances on prior low- and high-fidelity models.^9^–^15^ Our model has a remarkably simple rendering, and it is easy to assemble and replace components after multiple attempts. Using a durable 3D-printed anatomical chest model means we do not need to purchase animal parts or create resin molds; nor does the model degrade. Further, the design can be downscaled or upscaled depending on the size of model an individual wishes to practice on to allow mimicking large children, or small and large adults. Balloons can be prepped days in advance and can last in suspension for weeks at a time. Our model addresses complications created by buoyancy in a water-filled bath by careful positioning and using water-resistant tapes. While not initially offered as an option for our participants, our model could also withstand cannulation with a pericardiocentesis catheter set (Figure 3 [E–F]).

## LIMITATIONS

Despite these advances, there are several limitations to our model design. First, while the model components are easy to replace between attempts, we were unable to determine the duration of time nor the number of punctures our model could handle due to social-distancing safety measures. Secondly, during the USG pericardiocentesis simulations, the skin parchment paper would occasionally move when residents tried to puncture the material, which would also affect their ultrasonography. Finding a low-cost material that is replaceable, affordable, and ultrasound compatible would greatly improve the process. Real-time teaching feedback during the sessions was limited due to social distancing. This will be easily corrected when not conducting this learning opportunity during a pandemic. Also, while residents were asked whether the model was easier to use than prior models or cadavers, we did not ask what type of model had been used, their landmark or ultrasound approach, nor did we ask how long ago the procedure was performed. Lastly, free-rendering software, such as Blender, exists but will require time to learn through tutorials. Therefore, we provided the necessary files through a GrabCAD account for printing.

## CONCLUSION

During this current pandemic, low-cost, low-fidelity teaching models that do not require large groups, complex preparation, or in-person teaching are extremely valuable. Furthermore, low-occurrence, high-stress procedures often require cadaver models and repetition to develop provider competency. Therefore, our novel low-cost, low fidelity model offers an affordable resource that appropriately mimics human anatomy, provides easily replaceable components, and represents the environment while performing a pericardiocentesis by both a blind and ultrasound-guided approach.

**Population Health Research Capsule**

What do we already know about this issue?

*Pericardiocentesis models can be expensive; currently there are no low-fidelity models employing non-animal rib models*.

What was the research question?

*Is our model feasible to use, convenient, and does it provide competence in training?*

What was the major finding of the study?

*Over 90% of residents using the ultrasound and blind approach thought the model mimics a pericardial effusion*.

How does this improve population health?

*During the COVID-19 pandemic, these models provided an inexpensive workshop for a rare procedure that does not require large groups for learning*.

## Supplementary Information



## Figures and Tables

**Table 1:** Products used for the models including itemized costs.

Product	Number of units	Cost per unit ($)	Total cost ($)
Spinal needle 18G 3.50 in	2	3.52	7.04
10 mL syringe	2	0.18	0.36
Ultrasound Gel Aquasonic 100 Transmission	1	13.03	13.03
Duct tape 1.88 in x 45 yd	1	5.97	5.97
Clearlax polyethylene glycol 3350 850 g	1	22.49	22.49
7-quart graduated basin	2	2.20	4.40
22.8 cm latex balloons	5 bags of 20 balloons	1.50	7.50
Red, yellow, blue, green food coloring	4	0.93	3.69
Plastic wrap 100 Ft	1	2.19	2.19
Parchment paper roll sq ft	1	3.29	3.29
1.75mm filament 1 kg	1	22.99	22.99
¼″ yoga mat	1	14.99	14.99
Creality Ender 3 3D printer	1	179.99	179.99
Rhinoceros V6	1	195.00	195.00
Total cost			482.93

*in*, inches; *mL*, milliliters; *yd*, yards; *g*, gram; *cm*, centimeter; f*t*, foot; *kg*, kilogram; *3D*, three dimensional.
