# Supplementary material for: A Novel, Low-cost, Low-fidelity Pericardiocentesis Teaching Model
Source: West J Emerg Med. 2021 Jul 19;22(4):931–6. doi: 10.5811/westjem.2021.3.49876 (PMC8328165; doi:10.5811/westjem.2021.3.49876)
Supplement: Supplementary file 1 [file wjem-22-931-s001.docx]

**Supplemental**

The goal of this brief 2-4 minute survey is to examine perceptions regarding the effectiveness of our novel pericardiocentesis model.

Your survey response implies your consent to use the anonymous data.

I do consent to use of my anonymous data ________.

I do not consent to the use of my anonymous data _______.

Completion of the survey is encouraged but not required to use the pericardiocentesis model.

| Blind approach pericardiocentesis model | | | | | | | |
| --- | --- | --- | --- | --- | --- | --- | --- |
| Level of training | | PGY 1 | PGY 2 | PGY 3 | |  |  |
| Question | | Strongly agree | Agree | Neutral | Disagree | Strongly Disagree | Non applicable |
| Fidelity | | | | | | | |
| 1 | The model mimics a pericardial effusion | 5 | 4 | 3 | 2 | 1 | N/A |
| 2 | Ribs and rib spaces are palpable | 5 | 4 | 3 | 2 | 1 | N/A |
| 3 | Xiphoid process is palpable | 5 | 4 | 3 | 2 | 1 | N/A |
| 4 | Needle tip easily punctures pericardium | 5 | 4 | 3 | 2 | 1 | N/A |
| 5 | Aspiration of pericardial fluid is easy to accomplish | 5 | 4 | 3 | 2 | 1 | N/A |
| 6 | Confirmation of pericardial fluid aspirate is distinguishable from intracardiac aspirant | 5 | 4 | 3 | 2 | 1 | N/A |
| Convenience | | | | | | | |
| 1 | The pericardiocentesis model is easy to use | 5 | 4 | 3 | 2 | 1 | N/A |
| 2 | Replacing components is easy | 5 | 4 | 3 | 2 | 1 | N/A |
| 3 | This model is easier to use than other models | 5 | 4 | 3 | 2 | 1 | N/A |
| Competency | | | | | | | |
| 1 | The model helps develop accuracy with needle placement | 5 | 4 | 3 | 2 | 1 | N/A |
| 2 | The model helps with identifying important anatomical landmarks | 5 | 4 | 3 | 2 | 1 | N/A |
| 3 | The model provides real-time feedback | 5 | 4 | 3 | 2 | 1 | N/A |
| 4 | This model is adequate for training EM staff | 5 | 4 | 3 | 2 | 1 | N/A |
| 5 | This model increases competency in pericardiocentesis | 5 | 4 | 3 | 2 | 1 | N/A |

How many attempts were made to correctly aspirate the pericardial effusion? _________

When entering the pericardial sac did you aspirate blue or red dye first? ________

| US Guided Pericardiocentesis Model | | | | | | | |
| --- | --- | --- | --- | --- | --- | --- | --- |
| Level of training | | PGY 1 | PGY 2 | PGY 3 | |  |  |
| Question | | Strongly agree | Agree | Neutral | Disagree | Strongly Disagree | Non applicable |
| Fidelity | | | | | | | |
| 1 | The model mimics a pericardial effusion | 5 | 4 | 3 | 2 | 1 | N/A |
| 2 | Ribs are identifiable on US | 5 | 4 | 3 | 2 | 1 | N/A |
| 3 | Rib spaces are identifiable on US | 5 | 4 | 3 | 2 | 1 | N/A |
| 4 | Pericardial effusion is identifiable on ultrasound | 5 | 4 | 3 | 2 | 1 | N/A |
| 5 | Needle tip easily punctures pericardium | 5 | 4 | 3 | 2 | 1 | N/A |
| 6 | Aspiration of pericardial fluid is easy to accomplish | 5 | 4 | 3 | 2 | 1 | N/A |
| 7 | Confirmation of pericardial fluid aspirate is distinguishable from intracardiac aspirant | 5 | 4 | 3 | 2 | 1 | N/A |
| Convenience | | | | | | | |
| 1 | The pericardiocentesis model is easy to use | 5 | 4 | 3 | 2 | 1 | N/A |
| 2 | Replacing components is easy | 5 | 4 | 3 | 2 | 1 | N/A |
| 3 | This model is easier to use than other models | 5 | 4 | 3 | 2 | 1 | N/A |
| Competency | | | | | | | |
| 1 | The model helps develop accuracy with needle placement | 5 | 4 | 3 | 2 | 1 | N/A |
| 2 | The model helps with identifying important anatomical landmarks under ultrasound | 5 | 4 | 3 | 2 | 1 | N/A |
| 3 | The model provides real-time feedback | 5 | 4 | 3 | 2 | 1 | N/A |
| 4 | This model is adequate for training EM staff | 5 | 4 | 3 | 2 | 1 | N/A |
| 5 | This model increases competency in emergent bedside US guided pericardiocentesis | 5 | 4 | 3 | 2 | 1 | N/A |

How many attempts were made to correctly aspirate the pericardial effusion? _________

When entering the pericardial sack did you aspirate blue or red dye first? ________

Further comments:
